# Modified fasting therapy reduces blood pressure independent of weight loss, moderated by age, sex, and HbA1c: a retrospective mediation analysis in dysglycemic patients

**DOI:** 10.3389/fendo.2026.1976220

**Published:** 2026-10-07

**Authors:** Ziheng Ye, Jiapan Sun, Tingying Zhang, Jiahao Feng, Dongkai Zeng, Yidan Qin, Li Zhang, Jian Qin, Taoli Liu

**Affiliations:** Traditional Chinese Medicine Department, The Seventh Affiliated Hospital of Sun Yat-sen University, Shenzhen, China

**Keywords:** blood pressure, dysglycemia, fasting therapy, lifestyle intervention, mediation analysis

## Abstract

**Objective:**

To evaluate the effect of modified fasting therapy on blood pressure in dysglycemic adults and to examine the mediating role of weight loss and modifying effects of age, sex, and baseline glycemic status.

**Methods:**

This retrospective cohort study included 833 dysglycemic adults who underwent modified fasting therapy. Blood pressure, body weight, and laboratory parameters were measured before and after the intervention. Linear mixed models were used to estimate the effect of fasting on systolic blood pressure (SBP) and diastolic blood pressure (DBP), with adjustment for age, sex, baseline body mass index (BMI), glycosylated hemoglobin (HbA1c), and baseline blood pressure. Mediation analyses were performed using “MEMORE macro” and “wsMed” package to examine the mediating role of weight loss. Interaction terms were included to test effect modification by age, sex, and baseline HbA1c.

**Results:**

Fasting therapy significantly reduced SBP (−7.73 mmHg, 95% CI: −8.64 to −6.82, *p* < 0.001) and DBP (−4.76 mmHg, 95% CI: −5.39 to −4.12, *p* < 0.001). Mediation analyses revealed that weight loss did not significantly mediate the blood pressure-lowering effect for either SBP (indirect effect: −0.22, *p* = 0.588) or DBP (indirect effect: 0.09, *p* = 0.636). The fasting effect was significantly modified by age, sex, and baseline HbA1c, with greater reductions observed in younger patients, males, and those with higher HbA1c levels.

**Conclusions:**

Modified fasting therapy significantly reduces blood pressure in dysglycemic adults independent of weight loss. Age, sex, and baseline HbA1c modify the response, suggesting that personalized approaches may optimize the benefits of fasting-based interventions.

## 1 Introduction

Hypertension and dysglycemia are two closely intertwined metabolic disorders that frequently coexist, significantly increasing the risk of cardiovascular disease, stroke, and all-cause mortality (1–3). It is estimated that 66.3% patients with type 2 diabetes mellitus (T2DM) have concomitant hypertension (4), and the coexistence of these conditions substantially amplifies the burden of cardiovascular complications (5, 6). Importantly, this coexistence is not coincidental; insulin resistance and compensatory hyperinsulinemia are recognized as central pathophysiological links, promoting sodium retention, sympathetic nervous system overactivation, and vascular remodeling, thereby directly contributing to elevated blood pressure in dysglycemic individuals (7). Blood pressure therefore represents a core, modifiable component of the cardiometabolic risk profile in this population, rather than a separate disease endpoint. Despite the availability of various antihypertensive and glucose-lowering medications, achieving optimal blood pressure control remains a challenge in clinical practice (8), with a substantial proportion of patients failing to reach target levels (9, 10). This has prompted growing interest in lifestyle-based interventions as complementary or alternative strategies for blood pressure management, particularly in populations with metabolic abnormalities.

In recent years, fasting-based dietary interventions have gained considerable attention for their potential metabolic benefits, including weight reduction (11, 12), improved insulin sensitivity (13), and glycemic control (14). Emerging evidence suggests that fasting may also exert favorable effects on blood pressure regulation. Several randomized controlled trials have reported significant reductions in both systolic and diastolic blood pressure following various fasting regimens, including intermittent fasting (15), alternative-day fasting (16), and time-restricted eating (17). However, the underlying mechanisms by which fasting lowers blood pressure remain incompletely understood. While weight loss is commonly assumed to be the primary driver-given the well-established relationship between obesity and hypertension-the extent to which fasting-induced blood pressure reduction is mediated by weight loss, as opposed to other direct physiological effects, remains unclear. Notably, some studies have suggested that fasting may lower blood pressure independently of weight loss, potentially through mechanisms such as improved vascular endothelial function (18), and reduced oxidative stress level (19). However, definitive evidence from systematic mediation analyses is still lacking.

Furthermore, the blood pressure response to fasting may not uniform across all individuals. Patient characteristics such age, sex, and baseline glycemic status may modify the magnitude of the blood pressure-lowering effect, yet few studies have systematically examined these effect modifiers. Identifying which subgroups benefit most from fasting could facilitate personalized treatment recommendations and optimize clinical decision-making.

To address these knowledge gaps, we conducted a retrospective cohort study in dysglycemic adults who underwent a modified fasting therapy program. The aims of this study were: 1) to evaluate the effect of modified fasting therapy on blood pressure (both systolic and diastolic) in dysglycemic patients; 2) to examine whether the blood pressure-lowering effect is mediated by changes in body weight; and 3) to assess whether the effect is modified by age, sex, or baseline glycosylated hemoglobin (HbA1c) levels. We hypothesized that fasting therapy would significantly reduce blood pressure, but that this effect would be largely independent of weight loss, and that age, sex, and baseline glycemic status would significantly moderate the response.

In this study, we demonstrate that modified fasting therapy significantly reduces both systolic and diastolic blood pressure in dysglycemic adults, with the effect being largely independent of weight loss. Furthermore, we identify age, sex, and baseline HbA1c as significant moderators of the response, suggesting that personalized approaches may optimize the benefits of fasting-based interventions.

## 2 Methods

### 2.1 Study design and participants

This was a retrospective cohort study conducted at the seventh affiliated hospital of Sun Yat-sen university, Shenzhen, China. The study was approved by the ethics committee of the seventh affiliated hospital of Sun Yat-sen university (approval number: KY-2023-072-02). The requirement for informed consent was waived due to the retrospective nature of the study.

Electronic medical records of patients with dysglycemia who attended the Department of Traditional Chinese medicine between May 2018 and March 2026. Dysglycemia was defined as a diagnosis of T2DM or pre-diabetes according to the Guideline for the prevention and treatment of diabetes mellitus in China (2024 edition) (20). The diagnosis criteria of dysglycemia is based on measurements of fasting plasma glucose (FPG), glucose level 2 hours after oral glucose tolerance test (2h OGTT), and HbA1c, including T2DM (FPG ≥ 7mmol/L, 2h OGTT ≥ 11.1mmol/L or HbA1c ≥ 6.5mmol/L), impaired fasting glucose (IFG, 6.1 ≤ FPG < 7mmol/L), and impaired glucose tolerance (IGT, 7.8 ≤ 2h OGTT < 11.1mmol/L).

Patients were eligible for inclusion if they met the diagnosis of dysglycemia and had complete pre- and post-fasting measurements of blood pressure and body weight. Exclusion criteria included: 1) age < 18 years; 2) severe cardiovascular disease; 3) severe hepatic or renal dysfunction; and 4) incomplete baseline data on key covariates.

### 2.2 Intervention: modified fasting therapy

The modified fasting therapy protocol was delivered as an inpatient program under the supervision of skilled physicians at the Department of Traditional Chinese Medicine, the seventh affiliated hospital of Sun Yat-sen university. The modified fasting therapy protocol comprised three phases: 1) a pre-fasting phase, 2) a 5-day strict modified fasting phase, and 3) a food reintroduction phase. The pre-fasting phase included 1–2 “buffering days” during which only fruit was consumed. During the strict fasting phase, participants drank at least 3 L of water or non-caloric herbal tea daily and abstained from solid food. Daily energy intake was restricted to <550 kcal. After fasting, patients began a clear liquid diet, with food intake gradually increased to below standard dietary recommendations, followed by a slow transition to solid foods. L-carnitine supplementation was administered to promote fatty acid oxidation (21) and reduce hunger (22, 23). Patients received intravenous L-carnitine (2 g twice daily, Lee’s Pharmaceutical Holdings Limited, Hong Kong, China), from 9 a.m. on the pre-fasting day until 4 p.m. on the final day of fasting, to support fatty acid oxidation and reduce fasting-related hunger. For infusion, 2 g of L-carnitine was dissolved in 20 mL of saline.

### 2.3 Outcome measures

The primary outcomes were the change in SBP and DBP from baseline to the end of the fasting intervention period. Both SBP and DBP were measured using a validated automated device (Omron HEM-7120, Omron Corporation, Kyoto, Japan) after 10 minutes of seated rest, with the average of three readings used for analysis. Both outcomes were prespecified in the study protocol, and no hierarchy was established between them.

The secondary outcomes included body weight and body mass index (BMI) over the same period. Body weight was measured to the nearest 0.1 kg using a calibrated electronic scale with participants wearing light clothing and no shoes. Height was measured to the nearest 1 cm using a stadiometer. BMI was calculated as weight in kilograms divided by height in meters squared (kg/m^2^).

### 2.4 Laboratory measurements

Fasting blood samples were collected after an overnight fast of at least 10 hours and sent to the central accredited clinical laboratory, where all laboratory assays were performed using standardized protocols and quality control procedures.

### 2.5 Covariates

Baseline covariates included age (in years), sex (male/female), BMI (kg/m^2^), HbA1c (%), and baseline SBP/DBP (mmHg). These covariates were selected *a priori* based on clinical relevance and previous literature identifying them as potential confounders of the relationship between fasting therapy and blood pressure changes.

### 2.6 Mediator and effect modifiers

Change in body weight from baseline to end of intervention was prespecified as the mediator of interest for mediation analysis, based on the hypothesized pathway that fasting therapy may lower blood pressure through weight reduction. Age, sex, and baseline HbA1c were prespecified as potential effect modifiers, as they are known to influence both metabolic responses to dietary interventions and blood pressure regulation.

### 2.7 Statistical analysis

#### 2.7.1 Descriptive statistics

Baseline characteristics of all participants were summarized using descriptive statistics. Continuous variables with normal distribution were presented as mean ± standard deviation (SD), while non-normally distributed variables were presented as median with interquartile range (IQR). Categorical variables were presented as frequencies and percentages. Normality was assessed using the Shapiro-Wilk test and visual inspection of Q-Q plots.

#### 2.7.2 Longitudinal changes

Within-group changes from baseline to end of intervention for blood pressure, body weight, and other continuous variables were assessed using paired t-tests for normally distributed variables or Wilcoxon signed-rank tests for non-normally distributed variables. For the paired comparison, p-values were adjusted for multiple testing using the Benjamini-Hochberg procedure to control the false discovery rate as 5%. Both raw and adjusted p-values are reported.

#### 2.7.3 Primary analysis: linear mixed models

To evaluate the effect of modified fasting therapy on blood pressure over time, we fitted LMM using the “lmerTest” package in R, with restricted maximum likelihood (REML) estimation. The models included time (pre-fasting vs. post-fasting) as a fixed effect, with a random intercept for each participant to account for with-subject correlation of repeated measurements. The primary coefficient of interest was the time effect, representing the change in blood pressure from pre-fasting to post-fasting.

Covariates were selected *a priori* based on clinical plausibility and included age, sex, baseline BMI, baseline HbA1c, and baseline SBP (or DBP, depending on the outcome). Model assumptions were checked by inspecting residuals for normality and homoscedasticity.

#### 2.7.4 Secondary analysis 1: moderation analysis

To evaluate whether the effect of fasting on blood pressure was modified by patient baseline characteristics, we extended the LMM by including interaction terms: time × moderator. Prespecified moderators included age (continuous), sex (categorical), and baseline HbA1c (continuous). A significant interaction was interpreted as evidence that the moderator modified the effect of fasting on blood pressure. To visualize the interaction effects of moderators, marginal means were estimated using “emmeans” package in R, and line charts and histogram were generated to display the estimated changes in blood pressure with 95% confidence intervals across different levels of each moderator, providing an intuitive illustration of the interaction patterns.

#### 2.7.5 Secondary analysis 2: mediation analysis

Mediation analysis was performed to test whether the reduction in blood pressure was mediated by changes in body weight. For mediation analysis, we used the “MEMORE macro” in the SPSS without including the covariates, then validated the results from “MEMORE macro” by re-analyzing using the “wsMed” package in R with and without including covariates. The “wsMed” package is specifically designed for two-instance within-subject mediation analysis. The indirect effect (weight change pathway) was estimated using bootstrapping with 2000 resamples, and 95% confidence intervals were calculated. A bootstrapped confidence interval that did not include zero was considered evidence of significant mediation.

#### 2.7.6 Statistical software and significance

All statistical analyses were performed using SPSS version 25.0 (IBM Corp., Armonk, NY, USA) for descriptive statistics, longitudinal change, and mediation analysis; and R version 4.5.3 (R Foundation for Statistical Computing, Vienna, Austria) for mediation, LMM, and moderation analysis using the “wsMed” and “lmerTest” package. A two-sided p-value < 0.05 was considered statistically significant.

## 3 Results

### 3.1 Study population and data screening

A total of 6567 medical records of patients who underwent modified fasting therapy were initially screened. Among these, 5571 records were excluded due to normal glycometabolism, leaving 996 records of patients with dysglycemia for further assessment. After excluding 157 records with incomplete blood pressure measurements and 6 records of patients aged younger than 18 years, a final total of 833 records of dysglycemic adults were included in the present analysis. The detailed data screening process is illustrated in **Figure 1**.

**Figure 1:**
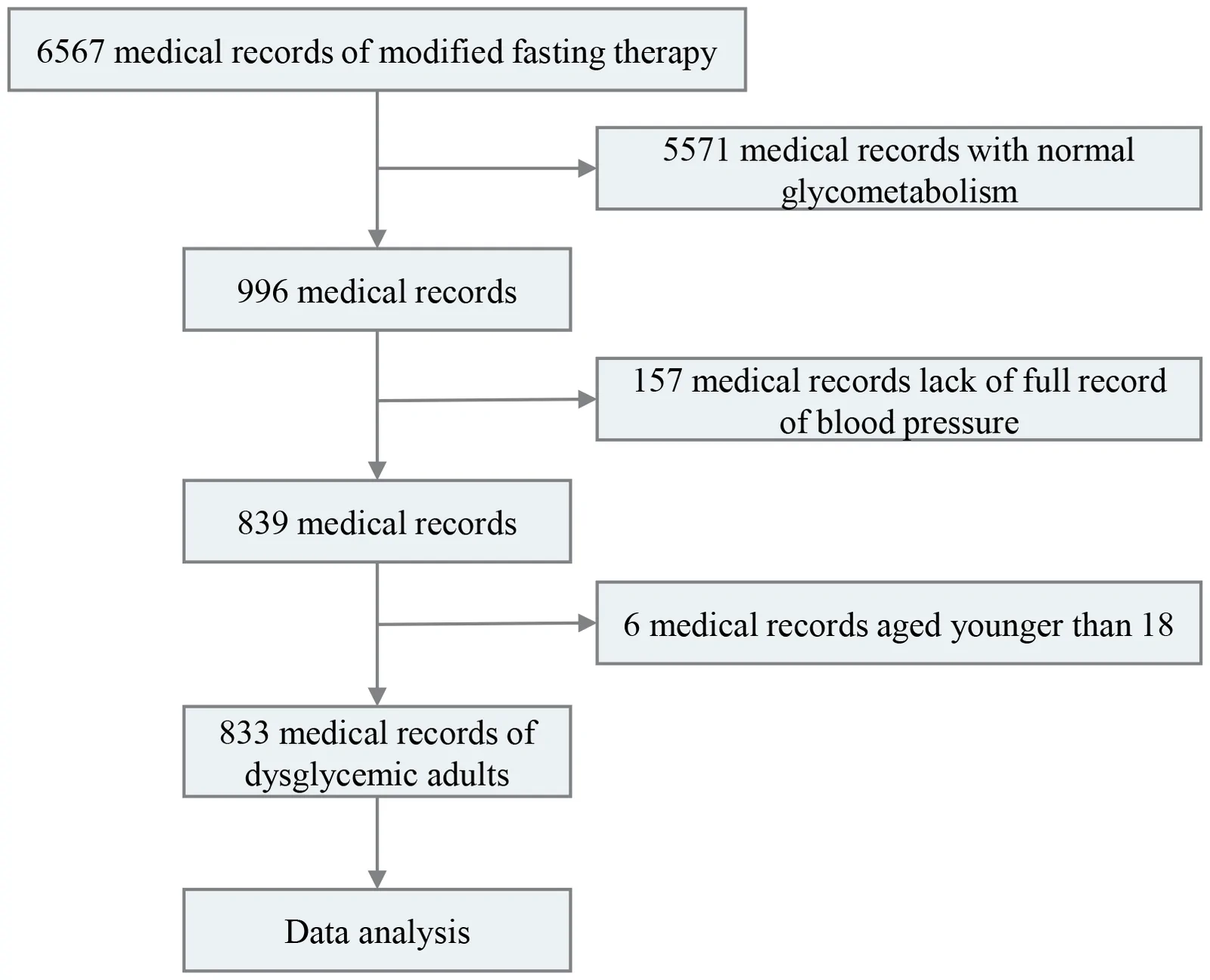
Flowchart of data screening of dysglycemic adults. Created in BioRender.

### 3.2 Baseline sociodemographic characteristics of the study population

A total of 833 participants were included in the final analysis. The median age of the study was 46 (14) years, with 46.7% (n=389) aged 18–45 years, 47.4% (n=395) aged 46–60 years, and 5.9% (n=49) aged over 60 years, as shown in **Table 1**. The cohort consisted of 443 (53.2%) male and 390 (46.8%) female participants.

**Table 1:** Baseline sociodemographic characteristics of study population.

Variable	n (%)
Sex	
Male	443 (53.2)
Female	390 (46.8)
Age (years)	
18-45	389 (46.7)
46-60	395 (47.4)
>60	49 (5.9)
Occupation	
Self-employed	65 (7.8)
Laborer	5 (0.6)
Civil servant	3 (0.4)
Farmer	1 (0.1)
Others	460 (55.2)
Enterprise manager	6 (0.7)
Retiree	62 (7.4)
Unemployed	11 (1.3)
Student	5 (0.6)
Office clerk	176 (21.1)
Professional technician	10 (1.2)
Freelancer	29 (3.5)
Smoking status	
Smoking	149 (17.9)
No smoking	684 (82.1)
Alcohol consumption	
Yes	144 (17.3)
No	689 (82.7)
Marriage	
Unmarried	95 (11.4)
Married	718 (86.2)
Divorced	18 (2.2)
Widowed	2 (0.2)

Regarding occupational status, the majority of participants were classified as “others” (55.2%, n=460), followed by office clerks (21.1%, n=176), self-employed individuals (7.8%, n=65), retirees (7.4%, n=62), freelancers (3.5%, n=29), and professional technicians (1.2%, n=10). Smaller proportions included enterprise managers (0.7%, n=6), laborers (0.6%, n=5), students (0.6%, n=5), civil servants (0.4%, n=3), and farmers (0.1%, n=1).

The prevalence of smoking was 17.9% (n=149), while alcohol consumption was reported by 17.3% (n=144) of participants. Regarding marital status, the majority were married (86.2%, n=718), followed by unmarried (11.4%, n=95), divorced (2.2%, n=18), and widowed (0.2%, n=2).

### 3.3 Baseline anthropometric and laboratory parameters of the study population

Baseline laboratory characteristics of the 833 participants are presented in **Table 2**. The median age of the cohort was 46 (14) years. Regarding anthropometric parameters, participants exhibited a median body weight of 79.4 (23.0) kg and a median BMI of 29.34 (6.41) kg/m^2^, indicating a predominantly overweight to obese population. Hemodynamic assessment showed elevated baseline blood pressure levels, with median SBP and DBP of 128 (23) mmHg and 84 (13) mmHg, respectively.

**Table 2:** Baseline anthropometric and laboratory parameters of the study population.

Variable	n	Value^a^
Age (years)	833	46 (14)
Body weight (kg)	833	79.4 (23.0)
BMI (kg/m^2^)	833	29.34 (6.41)
Abdominal circumference (cm)	132	98 (17)
SBP (mmHg)	833	128 (23)
DBP (mmHg)	833	84 (13)
FPG (mmol/L)	802	6.32 (2.11)
2h OGTT (mmol/L)	532	9.77 (5.13)
HbA1c (%)	748	6.8 (1.6)
INS (μU/mL)	629	10.18 (8.50)
FCP (ng/mL)	604	809.1 (536.5)
β-HB (mmol/L)	751	0.39 (1.13)
IGF-1 (ng/mL)	241	142 (68)
Cys-C (mg/L)	832	0.86 (0.22)
TG (mmol/L)	813	2.00 (1.65)
TC (mmol/L)	813	5.07 (1.46)
LDL-C (mmol/L)	813	2.92 (1.33)
HDL-C (mmol/L)	813	0.98 (0.30)
WBC (×10^9^/L)	828	6.78 (2.67)
RBC (×10^12^/L)	828	4.89 (1.00)
PLT (×10^9^/L)	823	245 (84)
CRP (mg/L)	461	2.44 (4.43)
TP (g/L)	833	71.7 (7.4)
ALB (g/L)	833	42.2 (4.4)
ALT (U/L)	833	29 (27)
AST (U/L)	833	21 (12)
GGT (U/L)	833	35 (36)
K (mmol/L)	833	4.06 (0.39)
Na (mmol/L)	833	139.7 (2.9)
Cl (mmol/L)	833	105.5 (3.0)

a Data are presented as median (IQR) for continuous variables. “n” represents the number of participants with available data for each variable.

BMI, body mass index; SBP, systolic blood pressure; DBP, diastolic blood pressure; FPG, fasting plasma glucose; 2h OGTT, glucose level 2 hours after oral glucose tolerance test; HbA1c, glycosylated hemoglobin; INS, insulin; FCP, fasting C-peptide; β-HB, beta hydroxybutyrate; IGF-1, insulin-like growth factor 1; Cys-C, cystatin C; TG, triglyceride; TC, total cholesterol; LDL-C, low density lipoprotein cholesterol; HDL-C, low density lipoprotein cholesterol; WBC, white blood cell; RBC, red blood cell; PLT, platelet; CRP, C-reactive protein; TP, total protein; ALB, albumin; ALT, alanine aminotransferase; AST, aspartate aminotransferase; GGT, gamma-glutamyl transpeptidase; K, potassium; Na, sodium; Cl, Chlorine;.

Glycemic parameters confirmed the dysglycemic nature of the cohort, with a median fasting plasma glucose of 6.32 (2.11) mmol/L and HbA1c of 6.8 (1.6) %. Among the 532 participants with available data, the median 2h OGTT was 9.77 (5.13) mmol/L. Lipid profiles at baseline included low density lipoprotein cholesterol (LDL-C) of 2.92 (1.33) mmol/L and triglyceride (TG) of 2.00 (1.65) mmol/L. Liver function showed median alanine aminotransferase (ALT), aspartate aminotransferase (AST), and gamma-glutamyl transpeptidase (GGT) levels of 29 U/L, 21 U/L, and 35 U/L, respectively. Other laboratory parameters, including serum electrolytes, blood count, and inflammatory markers, are detailed in **Table 2**. These findings collectively indicate that the study population was characterized by overweight/obesity, prehypertension, and dysglycemia at baseline.

### 3.4 Changes in anthropometric and laboratory parameters after fasting therapy

**Table 3** summarizes the changes in anthropometric, glycemic, and laboratory parameters following the fasting intervention. Significant improvements were observed in obesity-related measures: body weight decreased by 3.9 kg (95% CI: 3.8, 4.1) from 79.4 kg to 75.2 kg (*p* < 0.001), and BMI decreased by 1.47 kg/m^2^ (95% CI: 1.42, 1.52) from 29.34 to 27.89 kg/m^2^ (*p* < 0.001). Blood pressure also showed significant reductions, with SBP decreasing by 7 mmHg (95% CI: 6, 9) from 128 to 121 mmHg (*p* < 0.001) and DBP decreasing by 4 mmHg (95% CI: 3, 5) from 84 to 81 mmHg (*p* < 0.001).

**Table 3:** Changes in anthropometric and laboratory parameters after fasting therapy.

Variable	n	Pre-fasting^a^	Post-fasting^a^	Raw P	BH-adjusted P	Difference^ab^
Body weight (kg)	833	79.4 (23.0)	75.2 (21.4)	<0.001	<0.001	-3.9 (2.1)
BMI (kg/m^2^)	833	29.34 (6.41)	27.89 (6.10)	<0.001	<0.001	-1.47 (0.73)
Abdominal circumference (cm)	96	97.3 (17.0)	93.5 (15.2)	<0.001	<0.001	-3.7 (4.7)
SBP (mmHg)	833	128 (23)	121 (17)	<0.001	<0.001	-7 (19)
DBP (mmHg)	833	84 (13)	81 (13)	<0.001	<0.001	-4 (13)
FPG (mmol/L)	130	6.78 (1.98)	4.66 (1.99)	<0.001	<0.001	-1.71 (2.37)
INS (μU/mL)	139	11.15 (10.60)	5.12 (4.29)	<0.001	<0.001	-5.78 (7.01)
FCP (ng/mL)	112	810.3 (565.3)	439.7 (326.6)	<0.001	<0.001	-368.1 (311.5)
β-HB (mmol/L)	580	0.25 (0.76)	2.02 (1.82)	<0.001	<0.001	1.31 (1.71)
IGF-1 (ng/mL)	94	159 (67)	144 (74)	<0.001	<0.001	-13 (50)
Cys-C (mg/L)	786	0.86 (0.22)	0.80 (0.22)	<0.001	<0.001	-0.07 (0.12)
TG (mmol/L)	243	2.56 (2.39)	1.42 (0.78)	<0.001	<0.001	-0.94 (1.91)
TC (mmol/L)	242	5.24 (1.47)	5.32 (1.79)	0.04	0.047	0.15 (1.07)
LDL (mmol/L)	242	2.90 (1.23)	3.53 (1.33)	<0.001	<0.001	0.69 (1.08)
HDL (mmol/L)	242	0.97 (0.30)	0.85 (0.22)	<0.001	<0.001	-0.11 (0.23)
WBC (×10^9^/L)	811	6.8 (2.7)	5.5 (9.3)	0.73	0.73	-1.0 (10.0)
RBC (×10^12^/L)	810	4.89 (1.00)	4.53 (6.00)	0.19	0.20	-0.30 (6.77)
PLT (×10^9^/L)	63	270 (114)	281 (114)	0.18	0.20	2 (29)
CRP (mg/L)	95	3.07 (6.22)	4.06 (10.91)	0.003	0.004	0.44 (4.75)
TP (g/L)	793	71.7 (7.6)	71.5 (7.0)	0.22	0.23	0.1 (6.8)
ALB (g/L)	793	42.3 (4.3)	42.5 (3.9)	<0.001	<0.001	0.7 (3.8)
ALT (U/L)	798	29 (27)	31 (32)	<0.001	<0.001	1 (12)
AST (U/L)	798	21 (12)	27 (17)	<0.001	<0.001	4 (10)
GGT (U/L)	795	34 (35)	29 (26)	<0.001	<0.001	-5 (8)
K (mmol/L)	789	4.05 (0.38)	4.27 (0.40)	<0.001	<0.001	0.20 (0.47)
Na (mmol/L)	789	139.7 (2.9)	138.9 (2.7)	<0.001	<0.001	-1.0 (2.5)
Cl (mmol/L)	789	105.6 (3.0)	103.3 (3.0)	<0.001	<0.001	-2.0 (4.0)

a Data are presented as median (IQR).

b Differences were calculated as post-fasting minus pre-fasting values. P values were derived from paired Wilcoxon signed-rank tests.

Glycemic control improved markedly: FPG decreased by 1.71 mmol/L (95% CI: 1.37, 2.16) from 6.78 to 4.66 mmol/L (*p* < 0.001). Fasting insulin and C-peptide also showed substantial reductions (both *p* < 0.001). Beta-hydroxybutyrate (β-HB) increased significantly from 0.25 to 2.02 mmol/L (*p* < 0.001), confirming metabolic switching during fasting.

Lipid profiles demonstrated a favorable reduction in TG (Δ=-0.94 mmol/L, *p* < 0.001). However, modest increases were observed in total cholesterol (TC, Δ=0.15 mmol/L, *p* = 0.04), LDL-C (Δ=0.69 mmol/L, *p* < 0.001), and decrease in high density lipoprotein cholesterol (HDL-C, Δ=-0.11 mmol/L, *p* < 0.001). Modest changes in liver enzymes, electrolytes, and inflammatory markers were also observed (**Table 3**).

### 3.5 Primary analysis: linear mixed model

The results of the linear mixed models for changes in systolic and diastolic blood pressure are presented in **Tables 4** and **5**. After adjustment for age, sex, baseline BMI, HbA1c, and baseline blood pressure, fasting therapy was associated with a significant reduction in SBP of -7.73 mmHg (95% CI: -8.64, -6.82, *p* < 0.001) and DBP of -4.76 mmHg (95% CI: -5.39, -4.12, *p* < 0.001).

**Table 4:** Linear mixed model estimates for changes in blood pressure^a^.

Parameter	Estimate	Std. Error	P	95%CI	
				Lower bound	Upper bound
SBP					
Time (post-fasting vs. pre-fasting)	-7.73	0.47	<0.001	-8.64	-6.82
Age	0.07	0.02	0.003	0.02	0.12
Sex^b^	1.74	0.49	0.003	0.79	2.70
Baseline BMI	0.02	0.02	0.181	-0.01	0.06
HbA1c	-0.29	0.16	0.078	-0.60	0.03
Baseline SBP	0.67	0.01	<0.001	0.64	0.70
Interaction effects^c^					
Time × Age	0.13	0.05	0.006	0.04	0.22
Time × Sex	-2.17	0.94	0.021	-4.00	-0.33
Time × Baseline BMI	-0.04	0.03	0.197	-0.11	0.02
Time × HbA1c	-0.76	0.32	0.019	-1.39	-0.13
DBP					
Time (post-fasting vs. pre-fasting)	-4.76	0.32	<0.001	-5.39	-4.12
Age	0.03	0.02	0.038	0.002	0.065
Sex^b^	0.95	0.34	0.006	0.27	1.63
Baseline BMI	0.01	0.01	0.252	-0.01	0.04
HbA1c	-0.23	0.11	0.039	-0.45	-0.01
Baseline DBP	0.68	0.02	<0.001	0.65	0.71
Interaction effects^c^					
Time × Age	0.15	0.03	<0.001	0.09	0.21
Time × Sex	-2.99	0.64	<0.001	-4.24	-1.74
Time × Baseline BMI	-0.02	0.02	0.363	-0.07	0.02
Time × HbA1c	-0.76	0.22	<0.001	-1.19	-0.33

a Model adjusted for age, sex, baseline BMI, HbA1c, baseline pressure.

b Gender: 0=female, 1=male.

c Interaction terms were added in a separate model to test effect modification.

**Table 5:** Results of mediation analysis using MEMORE macro^a^.

Type of effect	SBP		DBP	
	Effect (95% CI)	P	Effect (95% CI)	P
Total effect	8.02 (6.99, 9.04)	<0.001	4.70 (4.01, 5.40)	<0.001
Direct effect	7.80 (6.60, 9.00)	<0.001	4.79 (3.98, 5.60)	<0.001
Indirect effect	0.22 (−0.74, 3.17)	–	−0.09 (−0.46, 1.12)	–

a Models not adjusted. Indirect effect was estimated using bootstrap resampling (2000 resamples).

Among the covariates, age (β=0.07, *p* = 0.003) and sex (β=1.74, *p* = 0.003) were significantly associated with SBP changes, while baseline HbA1c showed a marginal association (β=-0.29, *p* = 0.078). For DBP, age (β=0.03, *p* = 0.038), sex (β=0.95, *p* = 0.006), and HbA1c (β=0.29, *p* = 0.039) were all significant covariates.

### 3.6 Secondary analysis 1: moderation analysis

Interaction analyses revealed significant effect modification by age, sex, and baseline HbA1c for both SBP and DBP (**Table 4**). For SBP, Time × Age (β=0.13, *p* = 0.006), Time × Sex (β=-2.17, *p* = 0.021), and Time × HbA1c (β=-0.76, *p* = 0.019) were significant. For DBP, all three interactions were highly significant (all *p* < 0.001). No significant interactions were observed for Time × Baseline BMI in either model (*p*>0.05).

### 3.7 Visualization of interaction effects

As shown in **Figure 2**, the significant interaction effects of age, sex, and baseline HbA1c on the blood pressure-lowering effect of fasting were visualized. The sex-specific effects were shown in **Figures 2A, B**, indicating a greater reduction SBP in males (8.72 mmHg) than in females (6.55 mmHg), and DBP in males (6.13 mmHg) than in females (3.14 mmHg). The age-specific effects were visualized in **Figures 2C, D**, demonstrating a progressives decline in the magnitude of SBP and DBP reduction with increasing age (from 9.65 mmHg at age 30 to 5.82 mmHg at age 60, from 7.04 mmHg at age 30 to 2.45 mmHg at age 60, respectively). The HbA1c-specific effects were also displayed, showing an increasing trend in SBP and DBP reduction with higher baseline HbA1c levels (from 7.54 mmHg at 7% to 9.06 mmHg at 9%, from 4.54 mmHg at 7% to 6.05 mmHg at 9%, respectively). These visualizations corroborate the interaction analyses presented in **Table 4**, confirming that the blood pressure-lowering effect of fasting is significantly modified by age, sex, and baseline HbA1c as moderators.

**Figure 2:**
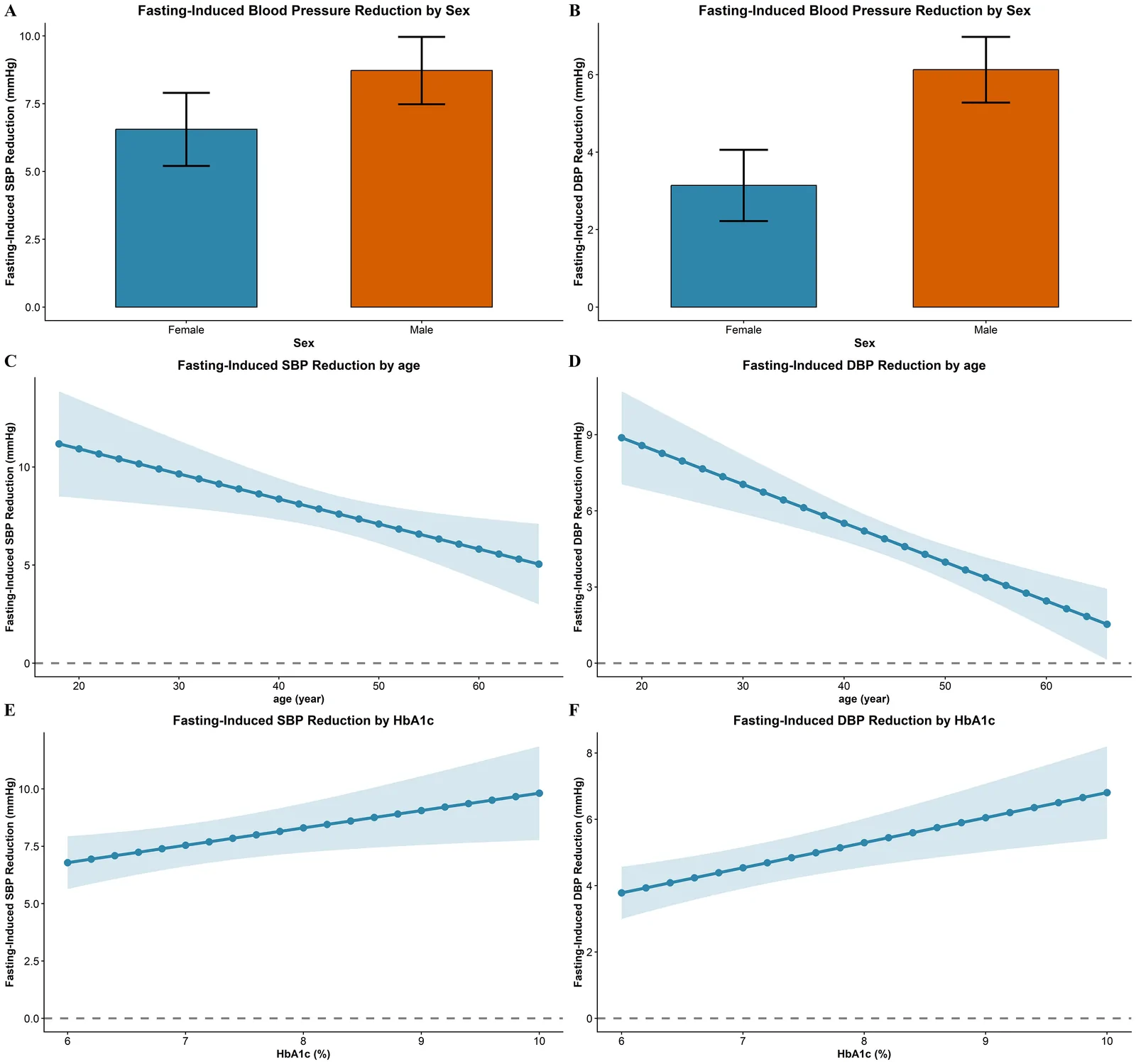
Visualization of interaction effects. Fasting-induced reduction of SBP **(A)** and DBP **(B)** by sex; Fasting-induced reduction of SBP **(C)** and DBP **(D)** by age; Fasting-induced reduction of SBP **(E)** and DBP **(F)** by baseline HbA1c.

### 3.8 Secondary analysis 2: mediation analysis

Mediation analysis was performed to examine whether the reduction in blood pressure following fasting therapy was mediated by changes in body weight. Analyses were conducted using the MEMORE macro (unadjusted) and the wsMed package (unadjusted and adjusted).

In the unadjusted MEMORE mediation analysis (**Table 5**), the indirect effect of weight change on the association between fasting and blood pressure reduction was not significant for either SBP (indirect effect: 0.22, 95% CI: -0.74-3.17) or DBP (indirect effect: -0.09, 95% CI: -0.46 to 1.12).

The wsMed mediation analysis was conducted to validate the results from MEMORE analysis (**Table 6**), showing a total effect of -8.02 (95% CI: -9.02 to -7.01, *p* < 0.001) for SBP, with a non-significant indirect effect of -0.22 (95% CI: -0.85 to 0.42, *p* = 0.588). For DBP, the total effect was -4.70 (95% CI: -5.38 to -4.02, *p* < 0.001), with a non-significant indirect effect of 0.09 (95% CI: -0.34 to 0.52, *p* = 0.636). After adjustment for age, sex, HbA1c, baseline BMI, and baseline blood pressure, the indirect effects remained non-significant for both SBP (-0.15, 95% CI: -0.71 to 0.40, *p* = 0.286) and DBP (0.30, 95% CI: -0.17 to 0.76, *p* = 0.361). These findings consistently demonstrate that the blood pressure-lowering effect of fasting is no mediated by weight loss, suggesting that alternative mechanisms are primarily responsible for the observed reductions in both SBP and DBP.

**Table 6:** Results of mediation analysis using “wsMed”.

Type of effect	Unadjusted		Adjusted^a^	
	Effect (95% CI)	P	Effect (95% CI)	P
SBP				
Total effect	-8.02 (-9.02, -7.01)	<0.001	-9.14 (-10.29, -7.99)	<0.001
Direct effect	-7.80 (-8.99, -6.61)	0.004	-8.99 (-10.27, -7.72)	<0.001
Indirect effect	-0.22 (−0.85, 0.42)	0.588	-0.15 (-0.70, 0.40)	0.286
DBP				
Total effect	-4.70 (-5.38, -4.02)	<0.001	-4.30 (-5.27, -3.33)	<0.001
Direct effect	-4.79 (-5.60, -3.99)	<0.001	-4.60 (-5.67, -3.52)	<0.001
Indirect effect	0.09 (-0.34, 0.52)	0.636	0.30 (-0.17, 0.76)	0.361

a Models adjusted for age, sex, HbA1c, baseline BMI and baseline SBP/DBP (depending on the outcome). Indirect effect was estimated using bootstrap resampling (2000 resamples).

## 4 Discussion

In this retrospective cohort study of 833 dysglycemic adults undergoing modified fasting therapy, we made several important observations. First, fasting therapy significantly reduced both systolic and diastolic blood pressure, with estimated reductions of 7.73 mmHg (95% CI: 6.82, 8.64) for SBP and 4.76 mmHg (95% CI: 4.12, 5.39) for DBP after adjustment for potential confounders. Second, mediation analyses using both MEMORE and wsMed approaches consistently demonstrated that weight loss did not significantly mediate the blood pressure-lowering effect of fasting, with indirect effects accounting for only 1.8% of the total effect for SBP and 0.2% for DBP. Third, we identified age, sex, and baseline HbA1c as significant moderators of the fasting-induced blood pressure reduction, with younger age, male sex, and higher baseline HbA1c being associated with greater blood pressure reductions. These findings collectively suggest that the blood pressure-lowering effect of fasting is largely independent of weight loss and is modulated by key patient characteristics, with important implications for personalized lifestyle intervention strategies.

The observed reductions in blood pressure, 7.73mmHg for SBP and 4.76 mmHg for DBP, are clinically meaningful. Epidemiological evidence has demonstrated that a 5-mmHg reduction in SBP is associated with approximately a 10% reduction in the risk of major cardiovascular events and a 13% reduction in the risk of stroke (24). The SBP reduction observed in our study therefore exceeds this clinically relevant threshold, suggesting that modified fasting therapy may confer meaningful cardiovascular risk reduction in dysglycemic patients. Notably, the magnitude of blood pressure reduction observed in our study is comparable to that achieved with first-line antihypertensive monotherapy (25), highlighting the potential of fasting as an effective non-pharmacological adjunct to blood pressure management.

Unexpectedly, our study revealed that weight loss does not mediate the blood pressure-lowering effect of fasting. Despite a significant reduction in body weight (-3.9 kg, *p* < 0.001) and BMI (-1.47 kg/m^2^, *p* < 0.001), the indirect effect of weight change on the association between fasting and blood pressure reduction was non-significant for both SBP (indirect effect: -0.22, 95% CI: -0.85, 0.42, *p* = 0.588) and DBP (indirect effect: 0.09, 95% CI: -0.34, 0.52, *p* = 0.636). This finding challenges the conventional assumption that weight loss is the primary mechanism through which dietary interventions lower blood pressure (26, 27).

Several potential mechanisms may explain this weight loss-independent blood pressure reduction. First, fasting has been shown to improve vascular endothelial function by activating autophagy of endothelial progenitor cells (18). Second, fasting may reduce sympathetic nervous system activity (28), which plays a central role in blood pressure regulation. Third, fasting is associated with reduced sodium intake and enhanced natriuresis, which may lower blood pressure through volume reduction (29). Fourth, fasting-induced improvements in insulin sensitivity may directly lower blood pressure, as insulin resistance is a well-established contributor to hypertension (30, 31). Finally, fasting may modulate the renin-angiotensin-aldosterone system (RAAS), potential through changes in angiotensin II levels or aldosterone secretion (32, 33).

It is important to note that intravenous L-carnitine was administered as part of the modified fasting therapy protocol. L-carnitine supplementation has been independently associated with modest reductions in bodyweight, BMI, and fat mass, particularly among individuals with overweight or obesity (34). Therefore, some of the observed weight loss in our study may be attributable to L-carnitine rather than fasting alone. However, this potential confounding would not alter our primary conclusion that weight loss does not mediate the blood pressure-lowering effect of fasting-if anything, the presence of L-carnitine would be expected to enhance weight loss, making the absence of mediation by weight loss even more notable. Future studies with a fasting-only control group (without L-carnitine) are needed to isolate the specific contributions of fasting and L-carnitine to both weight loss and blood pressure reduction.

The consistency of our mediation findings across two independent analytical approaches (MEMORE and wsMed) and across both SBP and DBP outcomes strengthens the robustness of this conclusion. These findings suggest that the cardiovascular benefits of fasting extend beyond weight reduction, and that fasting may exert direct effects on vascular physiology that are independent of changes in body weight.

Our interaction analyses revealed that the blood pressure-lowering effect of fasting was significantly modified by age, sex, and baseline HbA1c. The age-dependent attenuation of the fasting effect, with SBP reductions of 9.65 mmHg at age 30, 5.82 mmHg at age 60 and DBP reductions of 7.04 mmHg at age 30, 2.45 mmHg at age 60, suggests that younger patients derive greater blood pressure benefits from fasting. This may be attributable to age-related changes in vascular compliance, with older individuals exhibiting increased arterial stiffness that may limit the vasodilatory response to fasting (35, 36). Alternatively, younger patients may have greater metabolic flexibility and adherence to the fasting protocol.

Sex-specific effects were also observed, with males demonstrating greater SBP and DBP reductions than females (8.72 mmHg vs. 6.55 mmHg for SBP, interaction *p* = 0.021; 6.13 mmHg vs. 3.14 mmHg for DBP, interaction *p* < 0.001). This finding is consistent with previous studies suggesting sex differences in blood pressure regulation and response to dietary interventions (37, 38). Potential mechanisms include differences in adipose tissue distribution, hormonal profiles (particularly estrogen), and baseline sympathetic nerve activity between males and females (39, 40).

The observation that higher baseline HbA1c was associated with greater SBP reductions (7.54 mmHg at HbA1c=7%, 9.06 mmHg at HbA1c=9%; interaction *p* = 0.019) and DBP reductions (4.54 mmHg at HbA1c=7%, 6.05 mmHg at HbA1c=9%; interaction *p* < 0.001) is particularly noteworthy. This suggests that patients with poorer glycemic control may derive greater cardiovascular benefits from fasting, possibly because they have a more pronounced metabolic derangement that can be reversed through fasting-induced improvements in insulin sensitivity and glycemic control (41). This finding aligns with the concept of “high-risk, high-benefit” in lifestyle interventions and suggests that fasting may be particularly beneficial for patients with suboptimal glycemic control.

Our findings are consistent with several previous studies examining the effects of fasting on blood pressure. A recent meta-analysis of randomized controlled trials reported that fasting was associated with significant reductions in both SBP (mean difference: 4.88 mmHg) and DBP (mean difference: 5.10 mmHg) (27). Our observed reductions of 7.73 mmHg and 4.76 mmHg for SBP and DBP, respectively, are slightly larger than these pooled estimates, which may be attributable to differences in study populations, fasting protocols, or baseline characteristics.

Fasting therapy also significantly improved glycemic parameters, with median FPG, insulin (INS), and fasting C-peptide (FCP) all elevated at baseline and decreasing significantly after the intervention (all *p* < 0.001). This concurrent improvement in blood pressure and glycemic control suggests pleiotropic cardiometabolic benefits, supporting fasting as a comprehensive lifestyle intervention for dysglycemic patients. Notably, the above findings align with a growing body of evidence suggesting that the metabolic benefits of fasting extend beyond weight loss. Studies have reported improvements in insulin sensitivity, lipid profiles, and inflammatory markers that were not fully explained by weight reduction (19, 42, 43). Our study extends this literature by formally testing the mediating role of weight loss using rigorous mediation analytical approaches and demonstrating that the blood pressure-lowering effect is largely independent of weight loss.

C-reactive protein (CRP) and LDL-C increased modestly after fasting. The transient CRP rise may reflect an acute stress response rather than sustained inflammation (44, 45), while the LDL-C increase may result from cholesterol mobilization during weight loss. Notably, our prior follow-up data showed that LDL-C levels significantly declined at 3 and 6 months post-intervention (46). Similarly, although several other laboratory parameters, including albumin (ALB), showed statistically significant changes, the absolute magnitudes were small (e.g., ALB: +0.3 g/L) and may not be clinically meaningful; with a large sample size, even trivial differences can achieve conventional significance thresholds, and these findings should be interpreted in the context of effect size rather than P values alone. Given the short intervention duration, longer-term monitoring of lipid and inflammatory profiles is warranted.

Our findings have several important clinical implications. First, the significant and clinically meaningful blood pressure reduction observed with modified fasting therapy supports its role as an effective adjunctive strategy for blood pressure management in dysglycemic patients, in whom blood pressure is a core modifiable risk factor rather than a separate disease endpoint. Clinicians may consider recommending fasting-based interventions to patients with suboptimal blood pressure control, particularly those who are motivated to pursue lifestyle modification. Second, the lack of mediation by weight loss suggests that fasting may be beneficial even for patients who are unable to achieve significant weight reduction. This is particularly relevant for patients who have difficulty losing weight through conventional dietary approaches. The weight loss-independent effects of fasting on blood pressure may provide an additional rationale for recommending fasting to patients with hypertension, regardless of their weight status. Third, the identified effect modifiers (age, sex, and baseline HbA1c) may facilitate personalized treatment recommendations. Younger patients, males, and patients with higher baseline HbA1c may derive greater blood pressure benefits from fasting and may be particularly good candidates for this intervention. This personalized approach aligns with the broader movement toward precision medicine in lifestyle interventions.

This study has several strengths. First, the use of a comprehensive analytical framework integrating linear mixed models, mediation analysis, and interaction testing allowed us to simultaneously evaluate the magnitude, mechanism, and modifiers of the fasting-induced blood pressure reduction. Second, the cross-validation of mediation findings using two independent analytical approaches (MEMORE and wsMed) strengthened the robustness of our conclusions. Third, the separate analysis of SBP and DBP allowed us to identify outcome-specific patterns of mediation and moderation.

Several limitations should be acknowledged. First, the retrospective design with simultaneous measurement at only two time points limits causal inference and temporal precedence, rendering our mediation findings exploratory and warranting future prospective trials. Second, the single-arm pre/post design and single-center setting cannot separate fasting-specific effects from general hospitalization or caloric-restriction effects and may limit generalizability of our findings to other populations or settings. Third, we did not assess other potential mediators, such as changes in dietary sodium intake, physical activity, or medication adherence, which may also contribute to the blood pressure-lowering effect. Fourth, we did not assess long-term sustainability of the blood pressure reduction, as only short-term follow-up data were available. Finally, although we adjusted for a range of potential confounders, residual confounding cannot be entirely excluded.

## 5 Conclusion

In conclusion, this retrospective cohort study demonstrates that modified fasting therapy significantly reduces both systolic and diastolic blood pressure in dysglycemic adults, with the effect being largely independent of weight loss. Age, sex, and baseline HbA1c significantly modify the response, suggesting that personalized approaches may optimize the benefits of fasting-based interventions. These findings support the potential role of fasting as an effective adjunctive strategy for blood pressure management in dysglycemic patients and highlight the need for future prospective studies to confirm these observations and elucidate the underlying mechanisms.

## Data Availability

The raw data supporting the conclusions of this article will be made available by the authors, without undue reservation.
